# Targeting mTOR for cancer therapy

**DOI:** 10.1186/s13045-019-0754-1

**Published:** 2019-07-05

**Authors:** Hui Hua, Qingbin Kong, Hongying Zhang, Jiao Wang, Ting Luo, Yangfu Jiang

**Affiliations:** 1 State Key Laboratory of Biotherapy, Laboratory of Stem Cell Biology, National Clinical Research Center for Geriatrics, West China Hospital, Sichuan University, Chengdu, 610041 China; 2 Laboratory of Oncogene, Cancer Center, West China Hospital, Sichuan University, Chengdu, China; 3 School of Basic Medicine, Chengdu University of Traditional Chinese Medicine, Chengdu, China; 4 Cancer Center, West China Hospital, Sichuan University, Chengdu, China

**Keywords:** Cancer, Drug resistance, mTOR, Oncogene, Targeted therapy

## Abstract

Mechanistic target of rapamycin (mTOR) is a protein kinase regulating cell growth, survival, metabolism, and immunity. mTOR is usually assembled into several complexes such as mTOR complex 1/2 (mTORC1/2). In cooperation with raptor, rictor, LST8, and mSin1, key components in mTORC1 or mTORC2, mTOR catalyzes the phosphorylation of multiple targets such as ribosomal protein S6 kinase β-1 (S6K1), eukaryotic translation initiation factor 4E binding protein 1 (4E-BP1), Akt, protein kinase C (PKC), and type-I insulin-like growth factor receptor (IGF-IR), thereby regulating protein synthesis, nutrients metabolism, growth factor signaling, cell growth, and migration. Activation of mTOR promotes tumor growth and metastasis. Many mTOR inhibitors have been developed to treat cancer. While some of the mTOR inhibitors have been approved to treat human cancer, more mTOR inhibitors are being evaluated in clinical trials. Here, we update recent advances in exploring mTOR signaling and the development of mTOR inhibitors for cancer therapy. In addition, we discuss the mechanisms underlying the resistance to mTOR inhibitors in cancer cells.

## Introduction

The mechanistic target of rapamycin (mTOR) is a dual-specificity protein kinase phosphorylating serine/threonine as well as tyrosine residues [1]. Since the catalytic domain of mTOR resembles that of lipid kinases such as phosphoinositide 3-kinase (PI3K), mTOR is considered as an atypical protein kinase belonging to the PI3K-related kinase family [2]. As a core component of several distinct complexes including mTOR complex 1 (mTORC1), mTOR complex 2 (mTORC2), and a putative mTOR complex 3 (mTORC3), mTOR has critical roles in diverse biological processes, such as cell proliferation, survival, autophagy, metabolism, and immunity [2, 3]. While mTOR and mammalian lethal with SEC13 protein 8 (mLST8) are common members of both mTORC1 and mTORC2, regulatory-associated protein of mTOR (raptor), the 40 kDa proline-rich Akt substrate (PRAS40), and DEP domain-containing protein 6 (DEPTOR) are specific members of mTORC1 [1, 2]. Instead, rapamycin-insensitive companion of mTOR (rictor) and mammalian stress-activated protein kinase-interacting protein 1 (mSIN1 or MAPKAP1) are unique components in mTORC2 but not mTORC1 [1]. Another rapamycin-insensitive complex, mTORC3, consists of ETV7, mTOR, and other undefined components [3]. mTORC1 senses nutrients, growth factors, and cellular energy to orchestrate nucleotide, lipid, and protein synthesis; inhibit autophagy; and stimulate cell growth [2]. mTORC2 is not only regulated by growth factors, but also activates type I insulin-like growth factor receptor (IGF-IR) and insulin receptor (InsR) through the tyrosine kinase activity of mTOR [1]. Besides, mTORC2 regulates the actin polarization and endocytosis [4, 5].

The mTOR signaling pathway has critical roles in mammalian metabolism and physiology. The de-regulated activity of mTOR is involved in many pathophysiological conditions, such as aging, Alzheimer’s disease, diabetes, obesity, and cancer [2]. As a natural inhibitor of mTORC1, rapamycin is able to increase lifespan in mice [6, 7]. mTOR activity is frequently de-regulated in a variety of human cancers, such as breast, prostate, lung, liver, and renal carcinomas. Upregulation of mTOR signaling can promote tumor growth and progression through diverse mechanisms including the promotion of growth factor receptor signaling, angiogenesis, glyolytic metabolism, lipid metabolism, cancer cell migration, and suppression of autophagy [1, 2]. Hence, mTOR is a promising target for cancer therapy. In this review, we discuss the roles of mTOR in human cancer and the rationales and challenges for developing mTOR inhibitors to treat cancer.

## The assembly of mTOR complexes

**Fig. 1:**
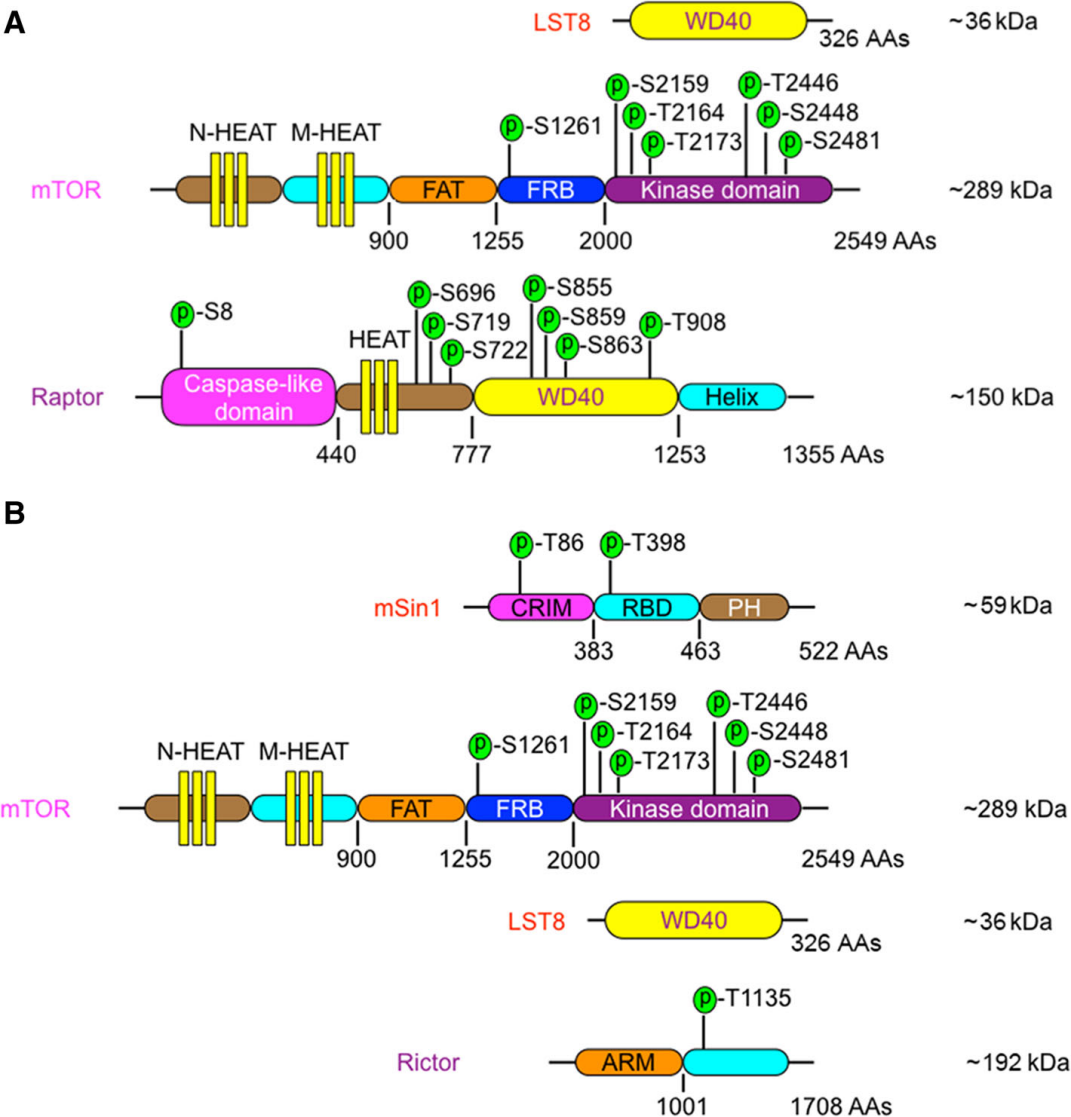
The domains in key components of mTORC1 and mTORC2. **a** The molecular weight, domains, and phosphorylation sites in key components of mTORC1, including mTOR, LST8, and raptor. **b** The molecular weight, domains, and phosphorylation sites in key components of mTORC2, including mTOR, mSin1, and rictor

The studies of mTORC1 structure demonstrate that mTORC1 adopts a dimeric architecture with an overall size of (280~300) × (200~210) × (100~130) Å^3^ [8, 9]. mTOR and LST8 form the core of mTOR complex that contains raptor and other regulatory proteins [8]. The human mTOR contains 2549 amino acids that form several domains including the NH_2_-terminal HEAT (N-HEAT), middle HEAT (M-HEAT), FAT, and kinase domain with a FRB insertion (Fig. 1). Raptor also contains a HEAT domain, as well as WD40 and caspase-like domain [8, 9]. Besides, LST8 has WD40 domain. The HEAT motifs have conserved Asp and Arg residues at positions 19 and 25, respectively. A signature motif of WD40 repeats is ~ 40 amino acids often ending with a tryptophan-aspartic acid (W-D) dipeptide [10]. The HEAT repeats 12–13 in one mTOR interact with the HEAT repeats 20–23 in the M-HEAT domain of another mTOR, thereby forming a dimer [8]. Raptor may stabilize the dimer by binding the HEAT repeats 11–13 in one mTOR and repeats 20–22 in another mTOR [8, 11]. In addition, raptor is required for recruiting substrates to mTORC1 [12, 13]. Both mTOR and raptor are subjected to phosphorylation at multiple residues (Fig. 1a), which positively or negatively regulates mTORC1 activity.

The assembly of mTORC2 and *Saccharomyces cerevisiae* TORC2 follows a similar principle to mTORC1. The human mTORC2 structure reveals a hollow rhombohedral fold with overall dimensions of ~ 220 × 200 × 130 (Å^3^) [14]. A dimer of mTOR is located in the core of this complex, while each mTOR or TOR heterodimerizes with rictor and mSIN1 [14, 15]. Rictor has an NH_2_-terminal armadillo (ARM) repeat cluster (~ 900 residues), and the rest of the rictor is largely unstructured (Fig. 1b) [16]. Interestingly, ARM and HEAT domains have similar conserved residues that form the hydrophobic domain core and may have a common phylogenetic origin [17]. In addition, mSin1 has a CRIM, a Ras-binding domain (RBD), and a pleckstrin homology (PH) domain [18]. During the assembly of mTORC2, the FRB domain of mTOR binds to mSin1 and the carboxy terminal region of rictor, while the NH_2_-terminal portion (residues 506–516) of rictor interacts with the COOH-terminal region (residues 1186-1218) of M-HEAT of mTOR [14]. In addition, mSin1 directly binds to rictor. Both rictor and mSin1 are responsible for recruiting substrates to mTORC2. Of note, both rictor and mSin1 have mTOR-independent partners. For example, rictor interacts with integrin-linked kinase and promotes its phosphorylation of Akt [19], while mSin1 interacts with Ras and inhibits ERK1/2 phosphorylation [20]. Thus, the outcome from the manipulation of rictor or mSin1 alone may not exactly reflect the function of mTORC2.

## Regulation of mTORC1 activity

The activity of mTORC1 is regulated by growth factors, cellular energy, stresses and nucleotides, etc. The lysosomes are primary sites for mTORC1 activation. The activation of mTORC1 by growth factors is dependent on Ras homolog enriched in the brain (RHEB), a lysosomal GTPase that directly interacts with mTOR and activates it [21]. Upon binding to growth factors such as epidermal growth factor (EGF) and insulin-like growth factor (IGF), the growth factor receptors (EGFR, IGFR, etc.) are activated, which in turn activate PI3K-PDK1-Akt signaling pathway. Active Akt phosphorylates tuberous sclerosis complex 2 (TSC2) and inhibits the TSC complex, a GTPase-activating protein (GAP) complex consisting of TSC1/2 and TRE2-BUB2-CDC16 domain family member 7 (TBC1D7) [22, 23]. The TSC complex can inactivate RHEB thereby inhibiting mTOR [24]. Therefore, the activation of Akt leads to the depression of RHEB and then activates mTORC1. Moreover, the ubiquitination of RHEB regulates its ability to activate mTORC1 [21]. The E3 ubiquitin ligase RNF152 catalyzes RHEB ubiquitination, leading to an increase in the interaction between RHEB and TSC [21]. In contrast, Akt can phosphorylate the deubiquitinase USP4 that promotes RHEB deubiquitination thereby releasing RHEB from TSC [21].

Downstream of the growth factor receptors, the mitogen-activated protein kinase (MAPK) also upregulates mTORC1 activity. Mechanistically, MEK1/2 promotes raptor phosphorylation through ERK1/2 and p90 ribosomal S6 kinase (RSK1/2). ERK1/2 directly phosphorylates raptor at S8, S696, and S863, while RSK1/2 phosphorylates raptor at S719/722 [25, 26]. Meanwhile, the intestinal cell kinase (ICK), a MAPK-related kinase, phosphorylates raptor at T908 [27]. Phosphorylation of raptor by ERK/RSK/ICK promotes the activation of mTORC1.

mTORC1 not only senses growth factors, but also responds to cellular energy. Low cellular energy results in an increase in AMP/ATP ratio, which activates the energy sensor AMP-dependent kinase (AMPK). AMPK stimulates the GAP activity of TSC and then promotes the inhibition of RHEB by TSC, leading to the downregulation of mTORC1 [28]. In addition, the TCA cycle metabolite ketoglutarate inhibits mTORC1 through repressing ATP synthase, increasing AMP/ATP ratio and activating AMPK [29]. Cellular energy deficiency usually leads to endoplasmic reticulum stress, which in turn induces the unfolded protein response (UPR). Ire1, ATF6, and PERK are three major mediators of the UPR. Upon ER stress, ATF6 can induce RHEB expression, which in turn promotes mTORC1 activation and cell survival [30]. However, overactivated mTORC1 is also harmful to cell survival under ER stress. Mutations in TSC1/2 or activation of RHEB renders cells hypersensitive to ER stress-induced apoptosis, which may be due to the downregulation of ATF4/6 by mTOR [31]. Therefore, mTORC1 may have versatile effects on cell survival under ER stress.

While the regulation of mTORC1 by growth factors is dependent on RHEB and the TSC complex, amino acids can stimulate mTORC1 independent of TSC. The regulation of mTORC1 by amino acids is very complicated, involving multiple amino acid sensors and protein machinery [32]. The lysosomal Ragulator (RAG) guanosine triphosphatases (GTPases) play key roles in the activation of mTORC1 by amino acids. RAGA or RAGB heterodimerizes with RAGC or RAGD [33]. Further, RAG proteins form a large complex with LAMTOR1/2/3/4/5, which recruit RAG and mTORC1 to the lysosomal surface [34]. The activity of RAG is regulated by two complexes, GATOR1 and GATOR2. GATOR1, which is composed of DEPDC5, NPRL2, and NPRL3, inhibits the GTPase-activated protein (GAP) activity of RAGA/B thereby repressing the activation of mTORC1 by amino acids [35]. Instead, GATOR2, a protein complex consisting of MIOS, WDR24, WDR59 SEH1L, and SECB, negatively regulates GATOR1 by inducing DEPDC5 degradation [35]. Furthermore, KICSTOR, a large complex consisting of KPTN, ITFG2, C12ORF66, and seizure threshold 2 (SZT2), recruits GATOR1 to the lysosomal surface and mediates the interaction between GATOR1 and RAG [36, 37].

Sestrin (SESN) is another category of negative inhibitors of amino acid-induced mTORC1 activation. Mechanistically, SESNs interact with GATOR2, leading to the release of GATOR1 from GATOR2. The released GATOR1 in turn inhibits RAG and mTORC1 [38–40]. Of note, SESN2 is known as a leucine sensor in mTORC1 signaling. Leucine directly binds to SESN2, leading to the dissociation of SESN2 from GATOR2. The released GATOR2 binds to GATOR1 and then prevents the inhibition of RAG by GATOR1. These sequential processes result in RAG-mediated mTORC1 activation [41]. To prevent the overactivation of mTORC1 by amino acids, there are negative feedback pathways to RAG-mediated mTORC1 activation. Two E3 ubiquitin ligases, RNF152 and SKP2, reportedly induce RAGA ubiquitination and potentiate the binding of RAGA to GATOR1 [42, 43]. While leucine sufficiency is sensed by SESN2, the stimulation of mTORC1 by arginine is mediated by SLC38A9 [44]. Moreover, the ubiquitin ligase TRAF6 can catalyze K63 ubiquitination of both Akt and mTOR thereby promoting the activation of Akt and mTORC1 by amino acids [45, 46].

In addition, mTOR may be activated by lipid and cholesterol. Fatty acid metabolism leads to the de novo synthesis of phosphatidic acid (PA), which stabilizes both mTORC1 and mTORC2 [47]. Moreover, cholesterol can stimulate mTORC1 activation and growth signaling. Mechanistically, SLC38A9 acts as a lysosomal cholesterol sensor to stimulate the activation of mTORC1 by RAG complex [48]. Recently, it was reported that mTORC1 is also responsive to the levels of purine nucleotides [49]. While adenylate stimulates mTORC1 by inhibiting TSC, guanylate downregulates RHEB and then inhibits mTORC1 [49]. The mechanisms underlying the regulation of TSC and RHEB by adenylate and guanylate remain to be known.

## Regulation of mTORC2 activity

Although mTORC1 and mTORC2 are distinct complexes, there is a crosstalk between these two complexes. On one hand, mTORC2 can activate IGF-IR-Akt axis thereby upregulating mTORC1 [1]. On the other hand, mTORC1 feeds back to inhibit mTORC2 via S6K1, one of the substrates of mTORC1. Once activated by mTORC1, S6K1 phosphorylates rictor and mSin1 on T1135 and T86/398, respectively, leading to the impairment of mTORC2 integrity [50–52].

While mTORC2 directly activates IGF-IR and InsR, receptor tyrosine kinases such as EGFR, PDGFR, and IGF-IR can activate mTORC2 via PI3K. Mechanistically, PI3K-induced PtdIns (3,4,5) P3 (PIP3) binds to the PH domain of mSin1 and then disables the inhibition of mTOR kinase domain by mSin1, thereby activating mTORC2 [18]. In addition, PI3K promotes the association of mTORC2 with ribosome, where mTORC2 is activated [53]. Therefore, mTORC2 also responds to growth factors. Notably, another study suggests that mTORC2 activity is localized in the plasma membrane, mitochondria, and endosomal vesicles, and the activity of mTORC2 via the mSin1-PH domain at the plasma membrane is PI3K- and growth factor-independent [54]. In addition, IKKα interacts with mTORC2 and enhances its kinase activity towards Akt [55]. These data suggest that the activation of mTORC2 involves multiple location and different mechanisms.

How does mTORC2 respond to cellular energy and nutrients? The energy sensor AMPK inhibits mTORC1 and then releases the suppression of mTORC2 by mTORC1, leading to the activation of mTORC2 [56]. Thus, upregulation of mTORC2 may help cells adapt to low levels of cellular energy. Moreover, mTORC2 is activated by glutamine starvation. Activated mTORC2 upregulates the expression and phosphorylation of glutamine:fructose-6-phosphate amidotransferase 1 (GFAT1), the rate-limiting enzyme of the hexosamine biosynthesis pathway (HBP) [57, 58]. A study of budding yeast demonstrates that the LKB1-ELM1-GIN4/HSL1 axis is required for coordinating TORC2 signaling to the changes in carbon source [59]. It remains to know if similar pathway works in human cancer cells.

Similar to mTORC1, mTORC2 is also stabilized by phosphatidic acid (PA), a central metabolite in the synthesis of membrane phospholipids [60]. The generation of PA is catalyzed by the phospholipase D, diacylglycerol kinases, and lysophosphatidic acid acyltransferases. Moreover, the activity of mTORC1 and mTORC2 is regulated by mLST8 ubiquitination. It has been reported that the E3 ubiquitin ligase TRAF2 positively regulates K63-linked polyubiquitination of mLST8, which impairs its interaction with mSin1 and compromises the mTORC2 integrity, but enhances the assembly of mTORC1 [61]. On the contrary, the deubiquitinase OTUDB7 removes polyubiquitin chains from G_β_L to promote G_β_L interaction with mSin1 and the integrity of mTORC2 [61]. Besides, the exchange factor found in platelets, leukemic, and neuronal tissues (XPLN) interacts with mTORC2 and negatively regulates mTORC2 activity [62]. Lastly, mTOR is a target of proteasomal degradation when it is ubiquitinated by FBXW7 [63].

## Targets of mTORC1 and mTORC2

As a protein kinase, mTOR catalyzes the phosphorylation of its targets and regulates their activity. mTORC1 and mTORC2 have different substrates. While the repertoire of mTOR substrates keeps increasing, there are more targets remaining to be identified. S6K1 and 4E-BP1 are two well-known mTORC1 targets. mTORC1 phosphorylates S6K1 at T389 and 4E-BP1 at multiple residues [64]. Phosphorylation of S6K1 by mTORC1 leads to increased protein and nucleotide synthesis. While 4E-BP1 is a negative regulator of 5′cap-dependent mRNA translation, phosphorylation of 4E-BP1 by mTORC1 induces its dissociation from eIF4E, thereby relieving its inhibition of protein synthesis [65]. To cope with increased protein synthesis, mTORC1 also promote ribosome biogenesis by inducing ribosomal RNA transcription. Mechanistically, mTORC1 may translocate to the nucleus, where it binds to ribosomal DNA promoter [66–68]. Nuclear mTOR also phosphorylates TFIIIC and Maf1, thereby promoting tRNA gene transcription [69]. In fact, nuclear mTOR regulates RNA polymerase 1/2/3-driven transcription. In addition, mTORC1 phosphorylates the E3 ubiquitin ligase SKP2 at S64 and then inhibits SKP2 ubiquitination and degradation [70]. Given that SKP2 promotes the degradation of many proteins, mTORC1 may regulate the turnover of SKP2 substrates indirectly. Thus, mTORC1 not only promotes protein synthesis, but also regulates protein degradation.

Following the identification of mTORC2, it was found that protein kinase C (PKC) α/β were the substrates of mTORC2 that regulates the actin cytoskeleton [4, 71]. Moreover, mTORC2 phosphorylates and activates other AGC kinases, such as serum and glucocorticoid-induced kinase (SGK) and Akt. mTORC2 phosphorylates Akt at S473, leading to allosteric activation of Akt in cooperation with the catalytic activation by PDK1, which phosphorylates Akt at T308 [72]. During the synthesis of nascent proteins, mTORC2 can co-translationally phosphorylate some polypeptides while they are attached to the ribosome. IGF2 mRNA-binding protein (IMP) is responsible for the splicing and translation of IGF2 mRNA. mTORC2 co-translationally phosphorylates IMP1 at S181 and then promotes IMP1 binding to the untranslated region of IGF2 mRNA and enables translational initiation by internal ribosomal entry [73]. mTORC2 not only enhances the production of IGF2 protein, but also phosphorylates and activates IGF-IR and insulin receptor [1]. In contrast to mTORC1’s activity as a ser/thr kinase, mTORC2 has tyrosine kinase activity towards IGF-IR/InsR [1].

## mTOR inhibitors for cancer therapy

The activity of mTOR is frequently upregulated in human cancer. The aberrant activation of mTOR in human cancer may be attributed to mTOR pathway-activating mutations, amplification, or overexpression of the components of mTOR complexes and mutations or loss of negative regulators of mTOR. PIK3CA mutations are frequently detected in human cancer. Activation of PI3K promotes both mTORC1 and mTORC2 activation. In addition, mutations in KRAS and BRAF may lead to mTORC1 activation. Especially, KRAS can directly bind to PIK3CA (p110α) and activates PI3K pathway, leading to mTOR activation [74]. mTOR-activating mutations are observed in kidney cancer. While mTOR activity is usually upregulated by growth factors and amino acids, activating mutations in mTOR may result in RAG- and RHEB-independent mTOR hyperactivation, thus loss of the dependency on growth factors and amino acids [75]. Point mutations in RHEB and GATOR1 were also detected in renal cancer and endometrial cancer [76]. RHEB1 is overexpressed in acute myeloid leukemia (AML) and promotes AML progression [77]. Whereas mTOR amplification is rare in human cancer, rictor amplification is detected in various kinds of cancer, such as breast cancer, gastric cancer, and liver cancer [78, 79]. Moreover, rictor is overexpressed in human cancers of the brain, breast, lung, gastric, colon, liver, and tongue [80, 81].

Given that mTOR has critical roles in tumor progression, mTOR inhibitors hold promise in cancer therapy. Indeed, rapamycin analogs (rapalog) have been approved for treating cancer in the clinic. In addition, many mTOR inhibitors with different mechanisms of action have been developed, some of which are undergoing clinical trials in variety types of human cancer.

### Rapalog

Rapamycin was originally identified as an antifungal, immunosuppressive, and antiproliferative agent. Later studies revealed that rapamycin binds to the 12 kDa FK506-binding protein (FKBP12) and then inhibits mTORC1 [82]. Since rapamycin has poor solubility and pharmacokinetics, it is not suitable for treating human cancer. So far, several water-soluble rapamycin analogs have been developed. For example, temsirolimus and everolimus exhibit tumor-suppressive effects in vivo. Both temsirolimus and everolimus have been used to treat advanced renal cell carcinoma (RCC) in the clinic. Moreover, everolimus is prescribed for treating pancreatic neuroendocrine tumors and advanced breast cancer [83]. Besides, there are many clinical trials to evaluate the efficacy of rapalogs in treating other types of human cancer, such as advanced gastric cancer, hepatocellular carcinoma, non-small cell lung cancer, endometrial cancer, and mantle cell lymphoma (clinicaltrials.gov).

Of particular note, the effect of rapalog monotherapy on solid tumors is modest in the clinic. The incomplete inhibition of mTOR by rapalogs may result in limited clinical success. On the other hand, inhibition of mTORC1 may lead to feedback activation of IGF-IR and Akt, which compromises the anti-cancer effect of rapalogs [1]. Taking into account the complexity of mTOR signaling networks, it is not hard to understand that the response to rapalogs varies in patients with cancer, such as metastatic RCC. It is desirable that there are biomarkers to predict the responses to mTOR inhibition. KRAS, BRAF, and TSC mutations are known as resistant markers for mTOR inhibitors, whereas PIK3CA mutations are sensitive marker [84, 85]. However, the roles of TSC1/2 and mTOR mutations in responding to rapalogs remain controversial. Although it has been reported that mutations in TSC1/2 and mTOR are more frequent in RCC patients who respond well to rapalogs, the majority of rapalog responders have no mutations in mTOR pathway, suggesting that other factors are also involved in rapalog sensitivity [86]. Notably, rapalogs usually arrest cell proliferation but does not induce apoptosis. Despite the initial response, tumors frequently develop resistance to these agents.

### ATP-competitive mTOR inhibitors

To more completely inhibit mTOR, a number of ATP-competitive mTOR inhibitors have been developed to target both mTORC1 and mTORC2. Tumors that are addicted to the mTOR signaling pathway may be sensitive to this kind of inhibitors. Unlike rapalogs, ATP-competitive mTOR inhibitors can not only arrest cell growth, but also induce apoptosis. MLN0128 (also called INK128, sapanisertib, TAK-228) is a pan-mTOR inhibitor that has potent in vitro and in vivo anti-tumor effects, and has underwent clinical trials for solid tumors such as bone and soft tissue sarcoma, breast cancer, and primary effusion lymphoma, a non-Hodgkin B cell lymphoma that usually results from infection of Kaposi sarcoma-associated herpesvirus [87–90]. MLN0128 also reduces tumor growth in CD44-high HCC xenografts and resensitizes HCC to sorafenib [91]. Of note, MLN0128 is an effective agent even in tumors that are resistant to rapamycin or chemotherapy. A recent study demonstrates that MLN0128 can overcome resistance to everolimus and reduce tumor size by 20% in *PIK3CA*-mutant colorectal cancers [92]. In addition, MLN0128 can induce tumor shrinkage in patient-derived xenograft model of pancreatic neuroendocrine tumors, even in everolimus-resistant tumors [93].

PP242 (Tokinib) is another selective ATP-competitive inhibitor of mTOR that has a promising anti-cancer activity over several cancer types, such as leukemia, gastric cancer, and colon cancer [94, 95]. Given that the Akt-mTOR signaling pathway is upregulated in platinum-resistant cancer cells, studies demonstrate that mTORC1/2 inhibitor, such as PP242 and MLN0128, can re-sensitize platinum-resistant ovarian cancer cells to carboplatin in vitro and in vivo [96, 97]. Mechanistically, mTOR inhibition leads to a sharp decrease in the translation of DNA damage and repair response and pro-survival mRNAs, including CHK1 [98]. Consistent with the inhibition of DNA repair, mTOR inhibitors are also effective in enhancing radiosensitivity or restoring radiosensitivity in radioresistant tumors [99, 100]. Moreover, inhibition of mTORC1/C2 signaling improves anti-leukemia efficacy of JAK/STAT blockade in CRLF2-rearranged and/or JAK-driven Philadelphia chromosome-like acute B cell lymphoblastic leukemia [101].

Both AZD2014 (vistusertib) and its analog AZD8055, two ATP-competitive mTORC1/2 inhibitors, are highly effective in treating estrogen receptor (ER)-positive breast cancer. Moreover, AZD2014 and AZD8055 can suppress breast cancer with acquired resistance to endocrine therapy, rapalogs, and paclitaxel [102, 103]. In addition, a combination of AZD2014 with paclitaxel reduces tumor volume in cisplatin-resistant ovarian cancer model [104]. Similar to PP242, AZD2014 enhances the radiosensitivity of glioblastoma stem-like cells [105]. Based on the above-described studies, it appears that the pan-mTORC1/2 inhibitors generally reverse rapalog resistance, endocrine resistance, chemoresistance, and radioresistance.

### Dual PI3K/mTOR inhibitors

Although inhibition of mTORC1 and mTORC2 can downregulate Akt S473 phosphorylation, mTOR inhibitors may paradoxically enhance the PI3K/PDK1 axis. Thus, an inhibitor targeting both PI3K and mTOR may have better anti-cancer activity compared to targeting mTOR alone [106, 107]. Due to the similarity between PI3K and mTOR, some chemicals can inhibit both PI3K and mTOR. NVP-BEZ235 (dactolisib) inhibits the activity of multiple class I PI3K isoforms, mTOR and ataxia telangiectasia, and Rad3-related protein (ATR) and has potent anti-cancer activity [108]. Notably, NVP-BEZ235 can penetrate the blood-brain barrier after systemic administration [109]. Therefore, it can be used to treat glioma and reverse temozolomide resistance [110]. In addition, NVP-BEZ235 can suppress paclitaxel-resistant gastric cancer, which exhibits increased PI3K/mTOR activity [111].

LY3023414, a complex fused imidazoquinolinone, is an oral PI3K/mTOR and DNA-PK inhibitor that has anti-tumor effects in animal models. Combination of LY3023414 with standard chemotherapeutic drugs has additive anti-tumor activity [112, 113]. Another dual PI3K/mTOR inhibitor voxtalisib (SAR245409, XL765), a pyridopyrimidinone derivative, significantly inhibits tumor growth in multiple human xenograft models [114]. Combination of voxtalisib and the MEK inhibitor pimasertib synergistically inhibits certain endometrial cancer cells growth [115]. Other dual PI3K/mTOR inhibitors include PQR309, XH00230381967, SN20229799306, GSK2126458 (omipalisib), and PKI-587.

Of note, PQR309 is a 4,6-dimorpholino-1,3,5-triazine-based, brain-penetrant, and orally bioavailable PI3K/mTOR inhibitor [116]. PQR309 effectively inhibits lymphoma in monotherapy and in combination therapy with other drugs, such as the BCL2 inhibitor venetoclax, the HDAC inhibitor panobinostat, the Bruton’s tyrosine kinase inhibitor ibrutinib, lenalidomide, the BET proteolysis-targeting chimera ARV-825, the proteasome inhibitor marizomib, and the anti-CD20 monoclonal antibody rituximab [117]. Moreover, PQR309 can suppress cancer cells with primary or secondary resistance to the PI3Kδ. PQR620 and the PI3K/mTORC1/2 inhibitor PQR530 effectively cross the blood-brain barrier [118].

The dual specificity PI3K/mTOR inhibitor gedatolisib (PKI-587, PF05212384) is a bis(morpholino-1,3,5-triazine) derivative [119]. Gedatolisib inhibits tumor growth in breast, colon, lung, and glioma xenograft models and displays efficacy against T cell acute lymphoblastic leukemia (T-ALL) and Philadelphia chromosome (Ph)-like B cell acute lymphoblastic leukemia (Ph-like ALL) [107, 120]. Combination of gedatolisib with ruxolitinib or dasatinib has superior efficacy than a single agent in CRLF2/JAK-mutant models and ABL/PDGFR-mutant models, respectively [120]. In addition, gedatolisib sensitizes head, neck, and nasophageal carcinoma to radiation therapy [121, 122] and sensitizes EGFR-resistant head and neck carcinoma to cetuximab [123]. Thus, gedatolisib may be a candidate sensitizer to radiotherapy and targeted therapy.

GSK2126458 (omipalisib) is an orally bioavailable inhibitor of PI3Kα and mTOR [124]. Omipalisib potently inhibits FGFR4-V550E tumor-derived cell and human rhabdomyosarcoma cell viability and reduces the growth of rhabdomyosarcoma in vivo [125]. In addition, a combination of the PI3K/mTOR inhibitor VS-5584 and the Wnt inhibitor ICG-001 synergistically inhibits AML with high PRL-3 expression [126]. Finally, the efficacy of mTOR inhibitor may be enhanced by linking the kinase inhibitor to rapamycin (RapaLink) [127]. EZH2 (Y641X)-mutant lymphomas show increased sensitivity to RapaLink-1 [128]. Given that RapaLink integrates the activity of both rapamycin and mTOR kinase inhibitor, it is worthwhile looking forward to the efficacy in clinical trials. Lastly, there are many drugs that may indirectly inhibit mTOR, such as aspirin and metformin [129–131].

## Principle mechanisms of mTOR inhibitor resistance in cancer

**Fig. 2:**
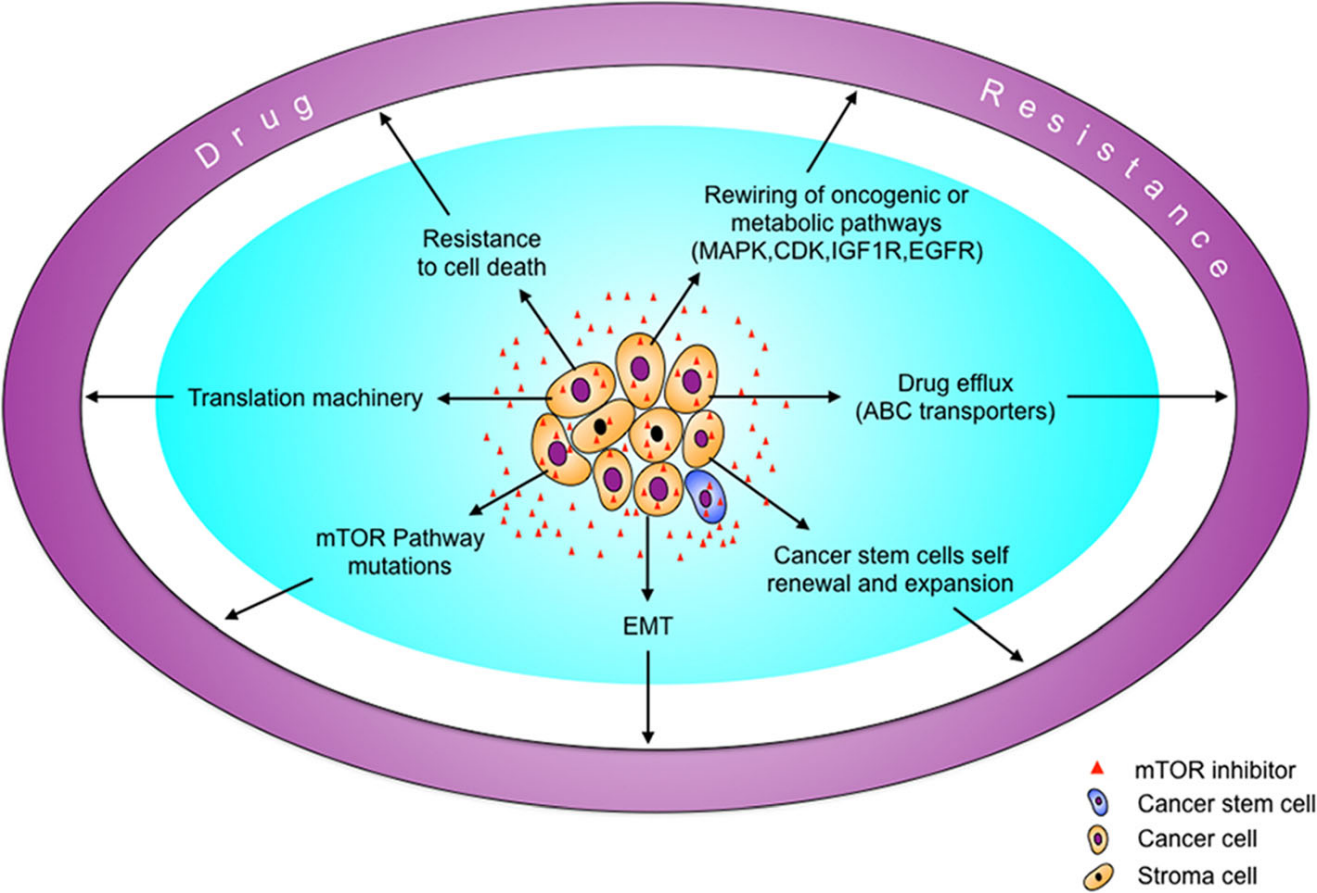
The mechanisms for resistance to mTOR inhibitors in cancer cells. ABC transporters, ATP binding cassette transporters; EMT, epithelial-mesenchymal transition

Drug resistance is a serious problem in treating cancer. Although there may be an initial response, long-lasting treatment with chemotherapeutic or molecular-targeted drugs often faces the challenge of drug resistance. Due to the tumor heterogeneity, some tumors do not respond to a given drug at all. Clonal selection, adaptive evolution, and resistance to cell death are general mechanisms for drug resistance. Due to the complexity and crosstalk in signaling networks, cancer cells may adapt to an inhibitor that targets a given signaling pathway via the compensatory activation of other pathways. Although mTOR inhibitors exhibit potent anti-cancer effects in many preclinical models, resistance does occur. As described below, there are multiple mechanisms underlying the resistance to mTOR inhibitors (Fig. 2).

### Drug efflux by ATP binding cassette transporters

ATP-binding cassette (ABC) transporters constitute drug efflux pumps that decrease the intracellular levels of drugs, leading to poor treatment outcome. Overexpression of ABC transporters is a general mechanism for multi-drug resistance in cancer. The same may be true for mTOR inhibitor resistance. In fact, the mTOR inhibitors rapamycin and NVP-BEZ235 are substrates of ABCB1 (P-glycoprotein) and ABCG2 (also called breast cancer resistance protein, BCRP), respectively [132]. In addition, AZD8055 is transported by both ABCB1 and ABCG2 [132].

Studies show that ABCB1 is overexpressed in luminal breast cancer cell lines that are resistant to everolimus [133]. Also, ABCB1 inhibits brain accumulation of everolimus [134]. Overexpression of ABCG2 in cancer cells confers significant resistance to PF-4989216, which can be reversed by an inhibitor or competitive substrate of ABCG2 [135]. Moreover, GDC-0980 is subject to active efflux by ABCB1 and BCRP, which limits its efficacy [136]. The affinity for ABC transporters may vary among different mTOR inhibitors. Lowering the affinity for ABC transporters or inhibiting ABC transporters may enhance the efficacy of mTOR inhibitors.

### Cancer stem cells

Cancer stem cells (CSCs) are a subpopulation in tumor mass that is extremely resistant to standard cancer therapy. Slow-cycling CSC is one of the major obstacles to eradicate tumor [137]. It is generally thought that the mTOR pathway is hyperactivated in CSC. Transforming growth factor-β (TGF-β) can induce epithelial-mesenchymal transition (EMT), which enhances cancer stem cell generation. mTOR is one of the mediators in TGF-β signaling pathways that enhances cancer stemness and drug resistance [138]. The inhibitory effect on CSCs has already been shown for some mTOR inhibitors [139]. Rapamycin, everolimus, and PF-04691502 suppress tamoxifen-induced activation of breast cancer stem cells [140]. Inhibition of mTOR restores tamoxifen resistance in breast cancer cells [141]. Moreover, the ATP-competitive mTOR inhibitor Torin1 and PI3K/mTOR inhibitor VS-5584 preferentially reduce CSC levels in multiple mouse xenograft models of human cancer [142, 143].

However, the interplay between mTOR inhibitors and CSC is complex. Previous studies show that expansion of CSC promotes the resistance to mTOR inhibitor in leiomyosarcoma [144]. PDK1 signaling toward PLK1-MYC activation leads to tumor-initiating cell activation and resistance to mTOR inhibition [145]. Inhibition of EZH2, a catalytic component of polycomb repressive complex which plays a critical role in stem cell maintenance, restores sensitivity to PI3K/mTOR pathway inhibition. It appears that the sensitivity to mTOR inhibitors in CSC may be context- or cell type-dependent. Of note, one study demonstrates that TP53 mutation and BCL2 phosphorylation affect the sensitivity of glioblastoma stem-like cells to mTOR inhibitor [146]. BCL2 (T56/S70) phosphorylation in TP53 wild-type glioblastoma stem-like cells is responsible for the lower sensitivity to the mTORC1/2 inhibitor AZD8055, as compared to TP53-mutated glioblastoma stem-like cells [146]. In addition, while mTOR inhibitors reportedly suppress CSC, one study demonstrates that treatment of TNBC cell lines with PI3K/mTOR inhibitor or TORC1/2 inhibitor expands CSC population through upregulating FGF1-FGFR-Notch1 axis [147]. Blocking FGFR or Notch1 may prevent resistance to TORC1/2 inhibitors by abrogating the expansion of drug-resistant CSCs in TNBC [49]. Moreover, another dual PI3K/mTOR inhibitor PF-04691502 can induce a stem cell-like gene expression signature in KRAS-mutant colorectal cancer models [148]. Together, these data suggest that the effects of mTOR inhibitors on CSC may be dependent on the genetic background and rewiring of cancer stemness pathways.

### Assembly of the translation machinery

Eukaryotic protein synthesis is regulated by several mechanisms including cap-dependent and cap-independent translation. The cap-dependent pathway involves many eukaryotic initiation factors (eIF), such as eIF1, eIF2, eIF3, eIF4A, eIF4B, eIF4E, eIF4H, eIF5, and eIF6. The protein synthesis is initiated by the association of the 40S ribosome subunit with eIF1A and eIF3, followed by binding of the eIF2-GTP-methionine tRNA complex to 40S subunit and then forming a 43S subunit [149]. The eIF4F complex, which consists of eIF4E, eIF4A, and eIF4G, binds to the m^7^G cap at the 5′ end of mRNA and then activates mRNA. The activated mRNA is recruited to the 43S complex and then subjected to ATP-dependent scanning of mRNA to locate the initiating AUG code [150]. Finally, the 60S ribosome subunit is associated with the 40S subunit to form the 80S initiation complex, possibly assisted by eIF5. For the initiation of cap-independent protein synthesis, the 40S ribosome subunit binds to an internal region of mRNA, which is referred to as internal ribosome entry sites (IRES), or the untranslated regions of mRNA.

Given that stimulation of cap-dependent translation is one of the major functions of mTORC1, the status of the translation machinery and modes of protein translation may impact on the efficacy of mTOR inhibitors. 4E-BPs are phosphorylated and inactivated by mTORC1. The sensitivity to PP242 is correlated with the extent to which 4E-BP1 phosphorylation is inhibited by this drug [151]. Loss of 4E-BPs in tumor cells results in the resistance to mTOR inhibition. The transcription factor Snail directly represses 4E-BP1 transcription and compromises the anti-cancer effects of mTOR inhibitors [152]. Of note, Snail is translationally regulated by eIF4E, which is exactly the target of 4E-BP. Phosphorylation of eIF4E (S209, etc.) promotes Snail synthesis [153]. Therefore, 4E-BP and eIF-4E can disable each other. Overexpression of eIF4E or phosphorylation of eIF4E (S209) by MAP kinase-interacting kinase 1 (Mnk1/2) leads to a shift from cap-dependent to cap-independent translation and then renders cancer cells insensitive to mTOR inhibition [154, 155]. Thus, inhibition of Mnk1/2 or its upstream kinase ERK1/2 may restore cap-dependent translation and the sensitivity of mTOR inhibitors [155]. On the other hand, inhibition of mTORC1 may lead to paradoxical phosphorylation of eIF4E in PI3K- and Mnk-dependent manner and promote cap-independent translation [156]. Hence, a combination of mTOR and Mnk inhibitors is an effective therapeutic strategy for cancer [157].

Notably, 4E-BP1 is not only phosphorylated by mTORC1, but also phosphorylated and inactivated by other kinases such as CDK1, CDK12, and GSK3 . CDK1 can substitute mTORC1 to phosphorylate 4E-BP1 and activate cap-dependent translation, which is resistant to mTOR inhibition [158]. In addition, CDK12 cooperates with mTORC1 to phosphorylate 4E-BP1 and releases it from mTORC1 target mRNAs thereby promoting their translation [159]. Therefore, combinatorial inhibition of mTOR and CDK1/12 may be synthetically lethal to cancer cells. Furthermore, GSK3β can directly phosphorylate4E-BP1 at the same residues (T37/46) that are phosphorylated by mTOR and CDK1 [160]. Given that mTORC2 positively regulates Akt, the negative regulator of GSK3β, mTOR kinase inhibitor may paradoxically activate GSK3. Hence, combinatorial inhibition of mTOR and GSK3β may synergistically suppress tumorigenesis. 

### mTOR mutations

Gene mutations may affect the sensitivity of a drug that targets the protein encoded by this gene. More than 30 activating mutations of mTOR have been reported in human cancer, such as L1460P, C1483F, E1799K, F1888L, T1977R, V2006I, V2046A, S2215Y, L2230V, E2388Q, I2500F, R2505P, and D2512H [127, 161]. Cancer cells that harbor a subset of those mutations, including C1483F, E1799K, and S2215Y, are hypersensitive to rapamycin, whereas three mutations (A2034V, F2018L, and S2035F) in the FRB domain of mTOR are associated with rapamycin resistance [162, 163]. While tumor cells with mutations in the kinase domain are still responsive to rapalogs [161], mutations in the kinase domain of mTOR, such as M2327I, S2215Y, L2230V, E2388Q, and V2046A, may be responsible for the resistance to the ATP-competitive inhibitor MLN0128 [127]. It remains to know whether activating mutations in the kinase domain of mTOR are responsible for the resistance to allosteric mTOR kinase inhibitors other than MLN0128. In addition, there are recurrent mutations in other mTOR pathway genes, such as *raptor*, *rictor*, and *RHEB* [163]. RHEB-Y35N mutant gains the function to activate mTORC1 [161]. It warrants further studies to clarify which cancer-associated mutations in raptor, rictor, and RHEB may be associated with mTOR inhibitors resistance.

### Rewiring of oncogenic or metabolic pathways

The sensitivity to mTOR inhibitors is regulated by other oncogenic pathways, such as PI3K, MAPK, AURKA, and NF-kB signaling [164, 165]. Both the Ras/MAPK and PI3K/Akt/mTOR pathways are tightly involved in tumorigenesis. While tumors with PIK3CA/PTEN mutations or Akt hyperactivation usually are sensitive to mTOR inhibitors, KRAS/BRAF mutations are predictive biomarkers of mTOR inhibitor resistance [148, 166–169]. In addition, mTOR inhibition may lead to the activation of the MEK-Erk pathway. Combination of RAF/MEK inhibitors and mTOR inhibitors may be a strategy to treat KRAS-mutated cancer [170, 171]. Besides, the activation of Erk in response to mTOR inhibition can be abrogated by the CDK4/6 inhibitor palbociclib [172]. Combination of CDK4/6 and mTOR inhibitors synergistically inhibits tumor growth [172, 173]. Alternatively, combined inhibition of wee1, a protein kinase that regulates the G2 checkpoint in the cell cycle, with mTOR inhibition may selectively treat RAS-mutated cancer [174]. Lastly, treatment with everolimus or AZD8055 increases epidermal growth factor receptor (EGFR) activation in tumor cells, leading to drug resistance [175].

Although PIK3CA-mutated cancer is usually sensitive to mTOR inhibition, activation of GSK3β in response to PI3K/mTOR inhibition may lead to the resistance to PI3K/mTOR inhibitors in PIK3CA-mutated cancer [176]. A recent study demonstrates that lung squamous cell carcinoma adapt to chronic mTOR inhibition through the GSK3α/β signaling pathway, which involves the metabolic reprogramming via increased glutaminolysis [177]. One study also reveals that glutaminase (GLS) and glutamate levels are elevated in glioblastoma after treating with mTOR inhibitor [178]. Treatment with GSK3 inhibitors or the glutaminase inhibitor effectively overcomes the resistance to mTOR inhibition [176–178]. Moreover, the activation of the purine salvage pathway due to increased expression of hypoxanthine phosphoribosyl transferase 1 leads to the resistance to the dual PI3K/mTOR inhibitor gedatolisib [179]. In fact, mTOR is tightly involved in purine metabolism. mTORC1 is not only activated by purine nucleobases or nucleosides [49], but also promotes purine synthesis by ATF4-mediated upregulation of the mitochondrial tetrahydrofolate (mTHF) cycle enzyme methylenetetrahydrofolate dehydrogenase 2 (MTHFD2) [180]. Moreover, mTORC1 promotes de novo pyrimidine biosynthesis by S6K1-mediated phosphorylation of carbamoyl-phosphate synthetase 2, aspartate transcarbamylase, and dihydroorotase (CAD) [181, 182]. Therefore, the increased expression of hypoxanthine phosphoribosyl transferase 1 may rescue the defect in purine synthesis due to mTOR inhibition and help cancer cells adapt to mTOR inhibition.

Another compensatory response to mTORC1 inhibition is the upregulation of transglutaminase 2, a multifunctional enzyme that is involved in cross-linking polypeptide chains with e-(c-glutamyl)-lysine, apoptosis, signal transduction, cell migration, cell adhesion, and extracellular matrix remodeling [183–185]. Inhibition of transglutaminase 2 potently sensitizes mTORC1-hyperactive cancer cells to rapamycin in vitro and in vivo [183]. Moreover, mitochondria homeostasis is critical for cell growth and survival. Mitochondrial hyperfusion is an adaptive response to mTOR inhibition. Mechanistically, the translation of mitochondrial fission process 1 (MTFP1) is suppressed by mTOR inhibitors, which eventually results in mitochondrial hyperfusion, a process that antagonizes apoptosis [186].

## Clinical testing of mTOR inhibitors

Given that preclinical studies demonstrate the anti-cancer efficacy of mTOR inhibitors alone or in combination with chemotherapy, radiotherapy, and targeted therapy, there are many completed or ongoing clinical trials to test the efficacy of mTOR inhibitors for treating various types of human cancer (Table 1). In general, most of mTOR inhibitors are well tolerated, while there are some common adverse effects including fatigue, rash, mucositis, and metabolic complications. mTOR inhibitors are associated with a significantly increased risk of hyperglycemia, hypertriglyceridemia, and hypercholesterolemia [187]. Other adverse events of everolimus are thrombocytopenia, anemia, nausea, and stomatitis [188]. Ridaforolimus is orally bioavailable and better tolerated in children than the adults [189]. Deforolimus was well tolerated and showed encouraging anti-tumor activity across a broad range of malignancies when administered intravenously, and a dose of 12.5 mg/day is being evaluated in phase II trials [190].

**Table 1:** Clinical evaluation of mTOR inhibitors

mTOR inhibitor	Category	Combination	Cancer type	Phase	Response	PFS (months)	OS (months)	Ref. or trial ID*
Everolimus (RAD001)	Rapalog	None	Thyroid cancer	2	No CR/PR; SD (> 24 weeks) 58%	9 (95% CI 4–14)	18 (95% CI 7–29)	200
Everolimus	Rapalog	Letrozole	Relapsed ER(+) high-grade ovarian cancer	2	CR 0; PR 16%; SD 37%	3.9 (95% CI 2.8–11); 3-month rate, 47%; 6-month rate, 32%	13; 6-month OS rate, 84%	209
Everolimus	Rapalog	Exemestrane	ER(+) locally advanced or metastatic breast cancer	3	CBR 33.4% vs 18% (control; placebo plus exemestrane)	6.93 (95% CI 6.44–8.05) vs 2.83 (95% CI 2.74–4.14) (placebo plus exemestrane)	30.98 (95% CI 27.96–34.56) vs control 26.05 (95% CI 22.57–33.08)	NCT00863655
Everolimus	Rapalog	None	Advanced neuroendocine tumor	3	Not available	11.04 (95% CI 8.41–13.86) vs placebo 4.6 (95% CI 3.06–5.49)	44.02 (95% CI 35.61–51.75) vs placebo 37.68 (95% CI 29.14–45.77)	NCT00510068
Everolimus	Rapalog	Rituximab	Diffuse large B cell lymphoma	2	ORR 38% (90% CI 21–56%); CR 3/24; PR 6/24	2.9 (90% CI 1.8–3.8)	8.6 (90% CI 4.9–16.3)	212 NCT00869999
MLN0128	ATP-competitive	Paclitaxel and trastuzumab	Advanced solid tumors	1	CR 0; PR 8/54; SD (> 6 months) 6/54	Not available	Not available	87 NCT01351350
AZD2014 (Vistusertib)	ATP-competitive	None	Metastatic clear cell renal cancer	2	Response rate 4% for AZD1024, 13% for everolimus Progressive disease 69% vs 13% for everolimus treatment	1.8 vs 4.6 for everolimus treatment	4.9 for AZD1024	203
Voxtalisib (SAR24540; XL765)	ATP-competitive	None	Relapsed or refractory non-Hodgkin lymphoma or chronic lymphocytic lymphoma	2	CR 8/164 (4.9%); PR 22/164 (13.4%); SD 55/164 (33.5%); ORR 18.3% (40.3% for follicular lymphoma)	1.9 for follicular lymphoma Overall progression-free rate at 24 weeks, 38·6% (95% CI 30·9–46·3)	Not available	202 NCT01403636
Gedatolisib (PKI-587; PF05212384)	ATP-competitive	None	Recurrent endometrial cancer	2	CR 1/38 (3%); PR 5/38 (13%); SD > 16 weeks, 24% (37% for stathmin-low cancer, 11% for stathmin-high cancer)	3.7 (95% CI 2–5.6) for stathmin-low cancer; 3 (95% CI 1.87–5.7) for stathmin-high cancer	Not available	204 NCT01420081

*CR* complete response, *CBR* clinical benefit rate, *ORR* overall response rate, *OS* overall survival, *PFS* progression-free survival, *PR* partial response, *SD* stable disease. *, Registration number in ClinicalTrials.gov

Moreover, MLN0028-treated patients may suffer from anorexia, dyspenea and macunopapular rash [191]. In clinical trials of solid tumors, the PI3K/mTOR inhibitor NVP-BEZ235 (twice daily) is poorly tolerated, which leads to treatment discontinuation in some patients and limits its efficacy in treating cancer [192, 193]. Apitolisib (GDC-0980), another dual pan-PI3K/mTOR inhibitor, also has grade 3–4 adverse effects and is less effective than everolimus [194]. GSK2126458 (GSK458) plus trametinib has poor tolerability, due to skin and gastrointestinal toxicities such as diarrhea [195]. Daily oral administration of PF-04691502 (8 mg/day) has adverse events including fatigue, nausea, vomiting, hyperglycemia, and rash [196]. The occurrence of the above-mentioned adverse effects following treatment with mTOR inhibitors may be due to the critical roles of mTOR in metabolism and immunity.

### mTOR inhibitors monotherapy

Everolimus has been approved by the FDA for the treatment of advanced renal cell carcinoma, pancreatic neuroendocrine tumors, and advanced breast cancer [83]. Everolimus significantly improves progression-free survival (PFS) among patients with progressive advanced pancreatic neuroendocrine tumors [197]. As registered in clinicaltrials.gov, there are more than 80 clinical trials for mTOR inhibitor monotherapy in cancer patients. A phase 2 trial of everolimus in patients with recurrent adult low-grade gliomas demonstrates a high degree of disease stability [198]. Moreover, everolimus has a promising effect in patients with heavily pretreated, relapsed, or refractory classical Hodgkin’s lymphoma, with an overall response rate (ORR) of 45.6%, a median PFS of 8 months, and a long-term response (≥ 12 months) rate of 12% [188]. Of note, everolimus exhibits clinical activity as the first-line monotherapy in a phase 2 clinical trial in 27 patients with advanced biliary tract cancer [199]. Another phase 2 clinical trial in 35 patients with thyroid cancer demonstrates that everolimus has clinical benefit in patients with advanced differentiated thyroid cancer [200]. Also, single-agent ridaforolimus has anti-tumor activity and acceptable tolerability in advanced endometrial cancer patients [201]. These observations need to be validated in a large scale of randomized clinical trials.

Based on a phase 2 trial in 167 patients, oral administration of the mTOR kinase inhibitor voxtalisib (50 mg, twice daily) exhibits a promising efficacy in patients with follicular lymphoma but limited efficacy in patients with mantle cell lymphoma, diffuse large B cell lymphoma, or chronic lymphocytic leukemia/small lymphocytic lymphoma [202]. Of note, serious adverse events occurred in 58.1% of patients [202]. In contrast, the clinical efficacy of MLN0128 in patients with metastatic castration-resistant prostate cancer is limited, possibly due to the dose reductions secondary to toxicity [191]. Although it is expected that mTOR kinase inhibitor may have superior efficacy than rapalogs, a randomized phase 2 trial in patients with metastatic clear cell renal cancer demonstrated that the PFS and OS of AZD2014 were less than that of everolimus [203]. While the PI3K/mTOR inhibitor NVP-BEZ235 is poorly tolerated in cancer patients, a clinical trial in patients with recurrent endometrial cancer demonstrated that weekly intravenous administration of another P3K/mTOR inhibitor gedatolisib achieved moderate anti-cancer activity with tolerable toxicity [204].

### mTOR inhibitors in combination therapy

While mTOR inhibitor monotherapy has efficacy in some type of cancer, preclinical studies demonstrate strong rationales for combinatorial treatment with mTOR inhibitors and other drugs. For example, inhibition of both Akt/mTOR and WNT/β-catenin pathways synergistically suppresses AML [205]. As registered in clinicaltrials.gov, there are many clinical trials to test the efficacy of mTOR inhibitors in combination with other molecular targeted or chemotherapeutic agents. For example, everolimus is combined with one or several chemotherapeutic agents, such as taxol, cisplatin, carboplatin, oxaliplatin, irinotecan, temozolomide, and gemcitabine.

The phase 3 BOLERO-2 trial in patients with ER-positive/HER2-negative advanced or metastatic breast cancer demonstrates that a combination of everolimus and the aromatase inhibitor exemestane significantly improves PFS, while the OS is not improved [206, 207]. Accordingly, a combination of everolimus and exemestane has been approved as a guideline for treating ER-positive/HER2-negative advanced or metastatic breast cancer [208]. In a phase 2 clinical trial, a combination of everolimus and the aromatase inhibitor letrozole achieved a 12-week PFS rate of 47% in patients with ER-positive relapsed high-grade ovarian cancer [209]. In addition, the combination of everolimus with trastuzumab and paclitaxel has a promising efficacy in patients with highly resistant HER2-positive advanced breast cancer (Table 1). This combination is currently under investigation in the BOLERO-1 phase 3 trial [210]. Moreover, a combination of everolimus with carboplatin is efficacious in treating metastatic triple-negative breast cancer, with a median PFS of 3 months (95% CI 1.6 to 4.6 months) and overall survival (OS) of 16.6 months [211]. In contrast, a combination of everolimus with gemcitabine/cisplatin has no synergistic effect in patients with metastatic triple-negative breast cancer. Hence, this combination still needs validation in more patients.

The CD20-targeted monoclonal antibody rituximab is a treatment for low-grade or follicular CD20-positive non-Hodgkin’s lymphoma. Diffuse large B cell lymphoma (DLBCL) is the most common type of non-Hodgkin’s lymphoma. A phase 2 study of everolimus (10 mg/day) in combination with rituximab demonstrated an overall response rate of 38%, a complete response rate of 12.5%, and a partial response rate of 25% among 24 patients with heavily pretreated DLBCL [212]. In addition, the combination of everolimus with rituximab or rituximab plus cyclophosphamide, doxorubicin, vincristine, and prednisone (R-CHOP) was well tolerated in DLBCL patients [212, 213]. It warrants further study to determine if the combination of everolimus with R-CHOP has a better response in patients with DLBCL. In addition, the combination of mTORC1/2 inhibitor with other targeted cancer drugs has been tested in clinical trials. Among 54 cancer patients who were treated with MLN0128 and trastuzumab/paclitaxel, 14.8% (8/54) of them achieved a partial response, and near 11% (6/54) cases had stable disease for more than 6 months [87]. According to a phase 1 trial (NCT02193633), the combination of paclitaxel and vistusertib is highly active and well tolerated in patients with high-grade serous ovarian cancer and squamous non-small cell lung cancer [214].

Given that IGF-IR signaling may induce mTORC1 inhibitor resistance, the combination of cixutumumab, a humanized monoclonal antibody against IGF-1R, and temsirolimus was tested in a clinical setting. This combination shows clinical activity in patients with sarcoma and adrenocortical carcinoma [215, 216]. In addition, a combination of everolimus (5 mg daily) and the multi-kinase inhibitor sorafenib (400 mg twice daily) exhibits anti-tumor activity in previously untreated patients with metastatic renal cell carcinoma with tolerable toxicity [217]. However, a combination of sorafenib and everolimus fails to achieve the target of 6 month PFS of 50% or greater among patients with unresectable high-grade osteosarcoma progressing after standard treatment [218]. For patients with recurrent glioblastoma, a combination of sorafenib (200 mg twice daily) and temsirolimus (20 mg weekly) is associated with considerable toxicity and poor efficacy [219].

In patients with metastatic castration-resistant prostate cancer, a combination of everolimus and the EGFR inhibitor gefitinib has no significant anti-tumor activity [220]. According to a phase 2 trial, a combination of sunitinib and everolimus as the first-line therapy exhibits poor efficacy in treating advanced renal cell carcinoma [221]. However, another phase 2 trial in patients with metastatic renal carcinoma demonstrates that the first-line sunitinib treatment followed by everolimus achieves a longer OS than the first-line everolimus followed by sunitinib, suggesting that the sequence may affect the outcome [222]. Moreover, a combination of imatinib and everolimus has limited activity in the treatment of patients with advanced chordoma [223]. The combination of pimasertib and voxtalisib showed a poor long-term tolerability and limited anti-tumor activity in patients with advanced solid tumors [224].

## Concluding remarks

The discovery of TOR in yeast and mTOR in mammals is a fundamental breakthrough in understanding cell and organism growth, metabolism, and diseases. In-depth studies to clarify the regulators and effectors of mTOR signaling have revealed multiple networks that work together to integrate growth factors, nutrients, sterols, and nucleotides signaling. The identification of the critical roles of mTOR and its regulators in tumorigenesis has driven the development of the ever-growing list of mTOR inhibitors. While some of the mTOR inhibitors have been approved to treat cancer patients, more mTOR inhibitors are under check to fulfill their promise for cancer therapy.

It appears that mTOR inhibitors have mixed efficacy in patients with distinct kinds of cancer and among patients with the same kind of cancer. Recent studies reveal that tumor organoids may help drug testing [225, 226]. Tumor organoids may be used to test the response of a given tumor to mTOR inhibitors. Alternatively, patient-derived tumor grafts may be transplanted to animals, followed by testing their response to mTOR inhibitors [227]. It would be of interest to determine if these emerging technologies are clinically relevant.

In the era of precise medicine, it needs to determine if there are predictive biomarkers that may guide the stratification of patients in clinical trials or help identify the patients who most likely benefit from treatment with mTOR inhibitors in a clinical setting. Gene testing is a promising approach to achieve this goal. The candidates for gene testing may include mTOR, PIK3CA, GATOR, KRAS, and BRAF. Mutations in PIK3CA and GATOR have been associated with higher sensitivity to mTOR inhibition in preclinical studies. Hence, PIK3CA mutations may be potential sensitive markers. In contrast, KRAS/BRAF mutations may be resistant biomarkers. Both DNA from tumor samples and ctDNA from the blood may be subject to testing of gene mutations. In addition, gene mutations in the tumors may be dynamic during cancer evolution or regression [228]. It remains to determine if dynamic testing of ctDNA during the course of therapy may monitor cancer evolution and better predict drug resistance, thereby adjusting the treatment regimen in time. Recent progress in liquid biopsy may help address this critical issue [229, 230]. In addition to gene testing, the solvable factors in the blood may be potential biomarkers as well. Of particular note, the mechanisms underlying the varied responsiveness to mTOR inhibitors in cancer patients may be complex. Rather than a single or few biomarkers, a set of biomarkers may be more powerful and accurate to meet the challenge.

Moreover, toxicity is a critical problem that precludes the clinical administration of drugs. Although mTOR inhibitors exhibit a promising efficacy in preclinical studies, some inhibitors have serious adverse effects in patients and have to be discontinued. Hence, elucidation of the mechanisms underlying these adverse effects may help manage them in the clinic.

Drug resistance is a serious challenge to successful cancer therapy. As discussed above, the mechanisms for mTOR inhibitor resistance are complex. Further studies to elucidate the diverse mechanisms may help design strategies to overcome the resistance to mTOR inhibition. Mechanism-based combination of mTOR inhibitors with chemotherapeutic agents or molecular-targeted drugs may be practical in the clinic. We expect the results from many ongoing clinical trials to validate the most powerful regimens that include mTOR inhibitors.

## Data Availability

Not applicable.

## Abbreviations

4E-BP1: Eukaryotic translation initiation factor 4E binding protein 1
DEPTOR: DEP domain-containing protein 6
IGF-IR: Type-I insulin-like growth factor receptor
MAPK: Mitogen-activated protein kinase
mLST8: Mammalian lethal with SEC13 protein 8
mSIN1: Mammalian stress-activated protein kinase-interacting protein 1
mTOR: Mechanistic target of rapamycin
PI3K: Phosphoinositide 3-kinase
PKC: Protein kinase C
PRAS40: 40 kDa proline-rich Akt substrate
Raptor: Regulatory-associated protein of mTOR
RCC: Renal cell carcinoma
RHEB: Ras homolog enriched in the brain
Rictor: Rapamycin-insensitive companion of mTOR
S6K1: Ribosomal protein S6 kinase β-1
TSC: Tuberous sclerosis complex
